# The Position of the Medical Profession in Society

**Published:** 1850-04

**Authors:** 


### THE POSITION OF THE MEDICAL PROFESSION IN SOCIETY.

This is the subject of the introductory lecture of Prof. Paul F. Eve to his class at the opening of the last session of the Medical College of Georgia, and full justice has the able and experienced teacher done to his interesting subject. Prof. Eve points with as much feeling as judgment to the causes which have prevented the medical profession from attaining its merited position and influence in society. The argument of his lecture is excellent, and its spirit and aim are worthy of all praise. It may be long before medicine triumphs over all the evils that environ it, and takes its proper rank among the high callings of men, but every such effort as this of the great Georgia surgeon must hasten the day of its triumph

